# Adsorption of gallic acid, propyl gallate and polyphenols from *Bryophyllum* extracts on activated carbon

**DOI:** 10.1038/s41598-019-51322-6

**Published:** 2019-10-15

**Authors:** Pascual García-Pérez, Sonia Losada-Barreiro, Pedro P. Gallego, Carlos Bravo-Díaz

**Affiliations:** 1 Plant Biology and Soil Science Dpt., Biology Faculty, University of Vigo, E-36310 Vigo, Spain; 2 Physical Chemistry Department, Chemistry Faculty, University of Vigo, E-36310 Vigo, Spain; 3 REQUIMTE-LAQV, Chemistry and Biochemistry Department. Science Faculty, University of Porto, 4169-007 Porto, Portugal

**Keywords:** Biosurfaces, Sustainability

## Abstract

The adsorption of gallic acid (GA) and propyl gallate (PG) on activated carbon (AC) was studied as a function of the AC mass and temperature. Clean first order behavior was obtained for at least three half-lives and the equilibrium was reached after ∼4 h contact time. An increase in the temperature (T = 20–40 °C) increases their adsorption rate constant values (*k*_1_) by 2.5 fold but has a negligible effect on the amount of antioxidant adsorbed per mass of AC at equilibrium. We also analyzed the adsorption process of polyphenols from *Bryophyllum* extracts and ca 100% of the total amount of the polyphenols in the extract were adsorbed when using 7 mg of AC. Results can be explained on the basis of the Freundlich isotherm but do not fit the Langmuir model. Results suggest that the combination of emerging *in vitro* plant culture technologies with adsorption on activated carbon can be successfully employed to remove important amounts of bioactive compounds from plant extracts by employing effective, sustainable and environmental friendly procedures.

## Introduction

The current trends in food consumption in developed countries reflect that naturalness is a crucial factor for the majority of consumers and it forces the development of novel strategies in the food processing industry. Moreover, consumers are more willing to accept food additives from natural sources, obtained by environmentally-friendly procedures, rather than synthetic products^1^.

In order to satisfy consumers’ demands, plants have been extensively exploited as a major source of bioactive compounds, derived from their secondary metabolism as a result of their defense and adaptation mechanisms, in both food and pharmaceutical industries (among others). In this sense, polyphenols have gained much attention in such fields because of their ubiquitous presence in the plant kingdom and their organoleptic and health-enhancing properties^2^. Specifically, these polyphenolic compounds have been described to exert a beneficial effect on the prevention of several chronic diseases thanks to their strong antioxidant activity against the oxidative stress, which is believed to make an important contribution to cancer, neurodegenerative and cardiovascular diseases, diabetes, etc^3^.

**Figure 1:**
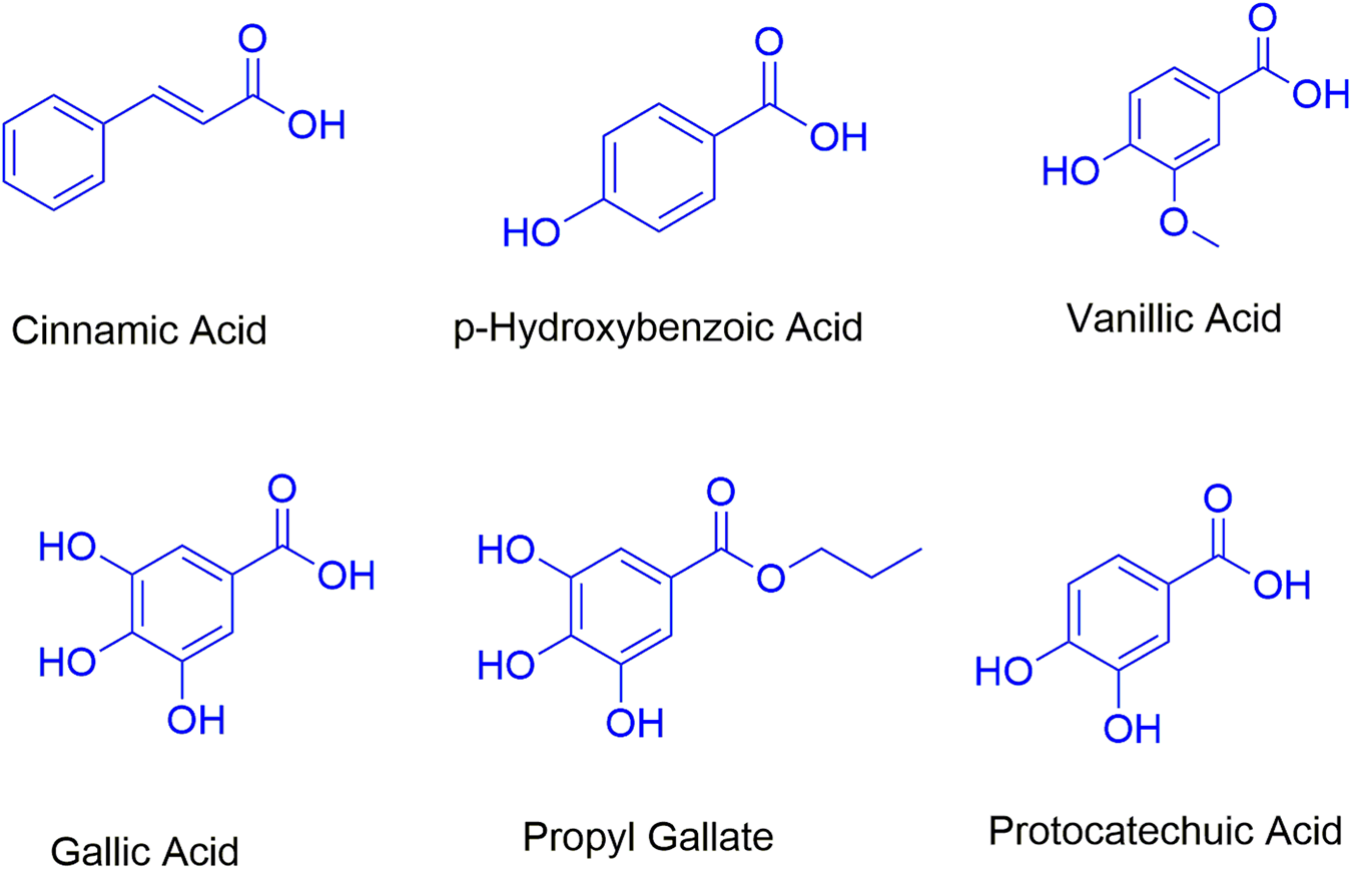
Chemical structures of some of the most important antioxidants present in *Bryophyllum* aqueous extracts^5^. Here we employed gallic acid and its propyl derivative.

Many plants have been studied for their novel antioxidants and, among others, *Bryophyllum* species have acquired much interest because of their high content of bioactive compounds (cinnamic acid, p-hydroxybenzoic acid, vanillic acid, gallic acid, protocatechuic acid, flavonoids^4,5^, Fig. 1). Their uses in traditional medicine, mainly in Africa, Asia and South America, can be highlighted where juice and leaf formulations have been employed for the treatment of several illnesses, such as insect bites, wounds, burns, fever, infections, coughs, arthritis, gastric ulcers, headache, diabetes and rheumatism^6,7^.

However, there are two major limitations when working with plant secondary metabolites:

1. Their small concentrations in plant, which involves the use of enormous amounts of fresh plant material. Besides, the plant breeding is not exempt from difficulties, since conventional farming is a long-term course, it is highly-sensitive and dependent on geoclimatic factors. Therefore, the rates of production of secondary metabolites can be limited^8^. Consequently, innovative applications are being progressively undertaken with the aim of overcoming the difficulties found in the traditional recovery of phenolic compounds from plant sources. Currently, *in vitro* plant tissue culture emerged as a solid methodology to avoid some of the obstacles attributed to conventional breeding, by offering a constant controlled production of true-to-type plant-derived products, under aseptic conditions, with total independency of the use of pesticides and climatic conditions^9^.
2. Polyphenols are, usually, extracted and purified with poor selectivity from plant sources. In addition, these processes generally, use large amounts of organic solvents that generate a negative impact on health and environment, making the whole process expensive and time-consuming^10^. As their use should be limited in as much as possible, new approaches for the recovery of polyphenols from plant extracts need to be developed.

Adsorption onto activated carbon (AC) is a well-established, economical and efficient method for the separation of the polyphenols from the complex aqueous solutions^11,12^. Activated carbon is a general term referred to any amorphous substance containing hydrophobic graphite-based layers with defined porosity and superficial heterogenic hydrophilic functional groups. AC generally proceeds from different low-cost natural sources including agricultural residues, forestry and industrial wastes and it is commonly used in adsorption processes for a plethora of environmental applications, e.g. wastewater treatment^13–16^.

As adsorbent agent, AC has the ability of extracting compounds from bulk solutions by attaching them by physical forces at its surface, keeping them chemically intact. This adsorption process is usually reversible to some extent, which implies that the adsorbed compound can be eventually recovered, and the AC is regenerated increasing its efficient re-usability. Among others, polyphenols are adsorbed at the surface of AC, and given their importance for the food and pharmaceutical industries, and studies on their adsorption processes have been widely reviewed^12^. However, limited studies have focused on the study of the adsorption of polyphenols from complex plant extracts using ACs and even less on their recovery after adsorption.

Here, we propose the combination of plant *in vitro* culture with the use of AC to face some of the limitations of traditional polyphenolic extraction with organic solvents by offering a low-cost, efficient and sustainable procedure in accordance with the current industrial needs and the increasing social awareness towards environmental conservation. We also carried out some preliminary experiments aimed to the recovery of adsorbed antioxidants (for example, by using NaOH and mixtures ethanol/water) but, the results are not reported because they are preliminary and the procedures need to be optimized.

Thus, the main aim of this work is two-fold. First, to establish an efficient plant *in vitro* culture and, second, to investigate some physicochemical aspects of the adsorption of polyphenols from *Bryophyllum* × *houghtonii* (*Bryophyllum daigremontianum* × *tubiflorum*) D. B. Ward aqueous extracts using AC. To gain some insights into the polyphenol adsorption process from plant extracts, we first investigated the adsorption of two commercial antioxidants, gallic acid (GA) and its derivative propyl gallate (PG). Their chemical structures are displayed in Fig. 1. We have chosen GA and PG because they are common phenolic compounds present in a variety of plant extracts including *Bryophyllum* species^5^. Besides, GA and PG constitute a set of two antioxidants (AOs) bearing the same reactive moiety but of different hydrophobicity, allowing us to analyze the effects of hydrophobicity of the adsorbate, adsorbent concentration and temperature on the adsorption process. Antioxidants with longer alkyl chains such as butyl or octyl gallates were not employed because their solubility in aqueous solutions is very low.

## Results and Discussion

### GA and PG adsorption kinetics: temperature effect

**Figure 2:**
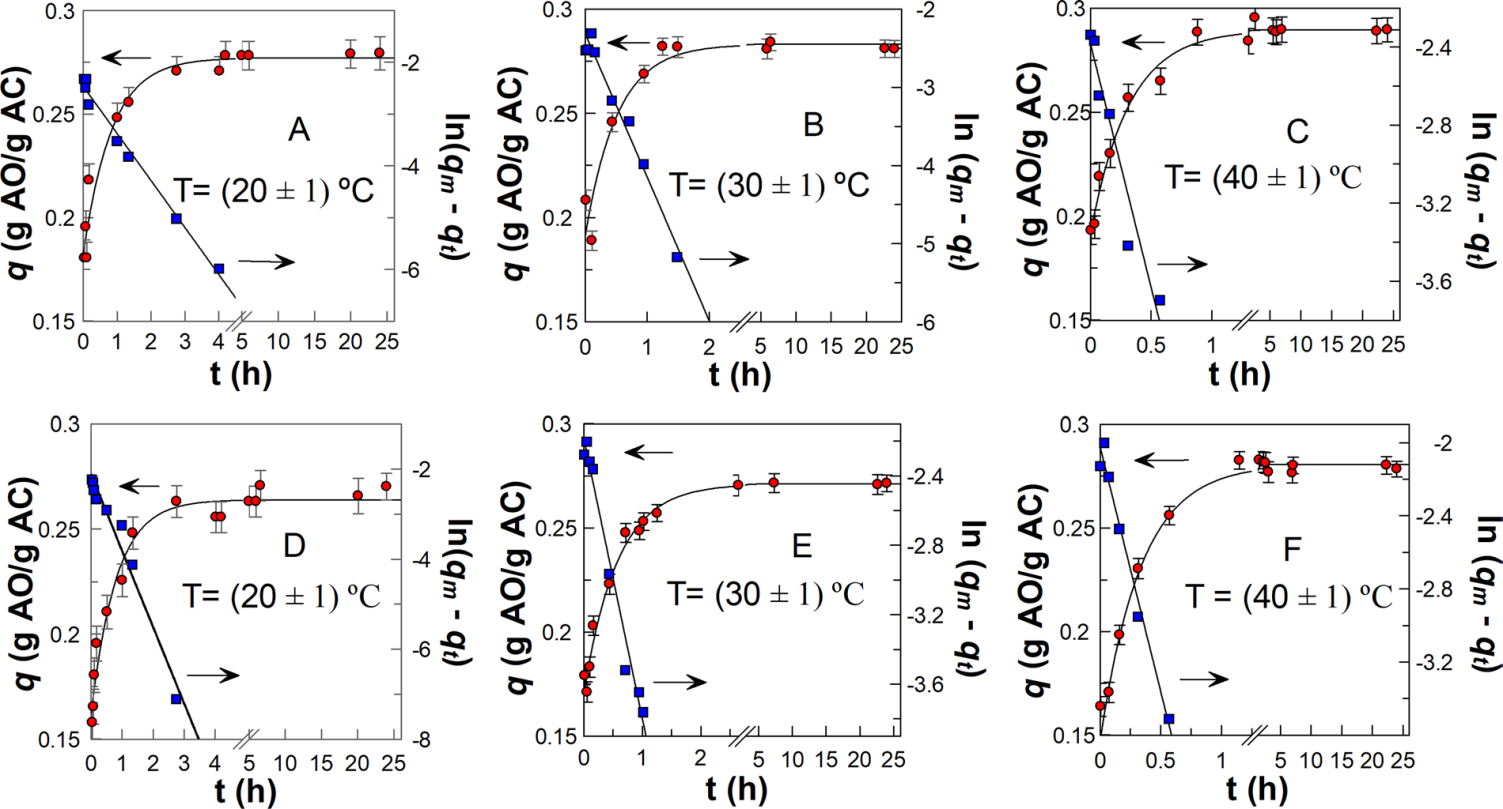
Variation of *q*, defined as the ratio between the amount (grams) of AOs adsorbed and the mass (grams) of AC at equilibrium (●) with the time, and linear plot (■) according to the first-order equation at the different temperatures. GA **(A–C)** and PG **(D–F)**, [AO]_initial_ = 0.20 g/L. *q*_*t*_ refers to the amount (g) of adsorbed AO at a time *t* per grams of AC and *q*_*m*_ is the maximum amount of antioxidant adsorbed per grams of AC. The solid lines are the theoretical curves obtained by fitting the experimental (*q*, time) or (ln (*q*_*m*_ − *q*_*t*_), time) pairs of data to the integrated first order equation, see experimental section.

Figure 2 shows typical kinetic plots illustrating the variation of the amount of antioxidant adsorbed per mass of AC at the equilibrium (*q*) with time for GA and PG. The corresponding first-order plots for at least three half-lives (with correlation coefficients >0.99) are also shown at the different temperatures. In all cases, the adsorption profiles reveal that equilibrium is reached after 4 h and it is maintained for, at least, 24 h, which was the time set for subsequent studies. The time needed to reach the adsorption equilibrium for GA is significantly lower than those reported by Soto *et al*.^17^ (15 h), Lehmann *et al*.^18^ (48 h) and by García-Araya *et al*.^19^ (150 h) under different experimental adsorption conditions, which might be attributable, among others, to variations in the physical properties of the sorbent.

The pseudo-first order adsorption constants (*k*_1_) and the maximum adsorption capacity, *q*_m_, for GA and PG at the different temperatures are shown in Table 1. The *k*_1_ values are temperature dependent increasing by a factor of 2.5 on going from T = (20 ± 1) °C to T = (40 ± 1) °C. The temperature dependence of *k*_1_ values stands in marked contrast to that of the maximum antioxidant adsorption, *q*_*m*_ values, which are nearly constant at the three temperatures with an average value of 0.284 ± 0.006 for GA and 0.273 ± 0.009 for PG.

**Table 1:** Kinetic parameters obtained by fitting (*q*, time) data to pseudo-first order equation for GA, PG and *Bryophyllum* extract at the different temperatures (*q*_*m*_ is the maximum amount of antioxidant adsorbed and *k*_1_ is the pseudo-first order adsorption constant). Measured temperatures are within ± 1 °C.

	T (°C)					
	20		30		40	
	*k*_1_ *(h*^*−1*^)	*q*_*m*_ *(g AO/g AC)*	*k*_1_ *(h*^*−1*^)	*q*_*m*_ *(g AO/g AC)*	*k*_1_ *(h*^*-1*^)	*q*_*m*_ *(g AO/g AC)*
Gallic Acid (GA)	1.19 ± 0.24	0.277 ± 0.003	1.89 ± 0.52	0.284 ± 0.006	3.16 ± 0.44	0.289 ± 0.002
Propyl Gallate (PG)	1.22 ± 0.19	0.264 ± 0.003	1.73 ± 0.26	0.272 ± 0.003	2.88 ± 0.49	0.282 ± 0.004
*Bryophyllum* extract	0.40 ± 0.03	0.124 ± 0.001	—	—	—	—

These values were essentially independent of nature of antioxidant (variations less than 4%) and this analysis provides insight into the adsorption mechanism, suggesting the phenolic ring as the cleavage site onto AC surface, regardless of the propyl ester moiety on phenolic ring of PG (Fig. 1)^12^.

### GA and PG adsorption isotherms: temperature effect

The effects of temperature (T = 20–40 °C) on the adsorption process of GA and PG were evaluated at different AC amounts. The experimental adsorption equilibrium data were fitted to two isotherm models, Langmuir and Freundlich equations and the goodness of fit was assessed.

### Langmuir isotherm

The Langmuir isotherm assumes a monolayer adsorption on homogeneous adsorbent surface with the same AO affinity in all the adsorption sites and no interaction among adsorbed antioxidants. The Langmuir isotherm can be described by the Eq. (1), where *K*_*L*_ is the equilibrium constant and *q*_*m*_ is the maximum adsorption capacity of the AC (g AO/g AC) and *C*_*e*_ the equilibrium concentration of the AO in the solution (g/L). Equation (2) is the reciprocal of Eq. (1) and it predicts that plots of 1*/q vs* 1/*C*_*e*_ should be linear and allows the determination of *K*_*L*_ and *q*_m_ parameters.

1 $$q=\frac{{q}_{m}{K}_{L}{C}_{e}}{1+{K}_{L}{C}_{e}}$$

2 $$\frac{1}{q}=\frac{1}{{q}_{m}{K}_{L}}\times \frac{1}{{C}_{e}}+\frac{1}{{q}_{m}}$$

**Figure 3:**
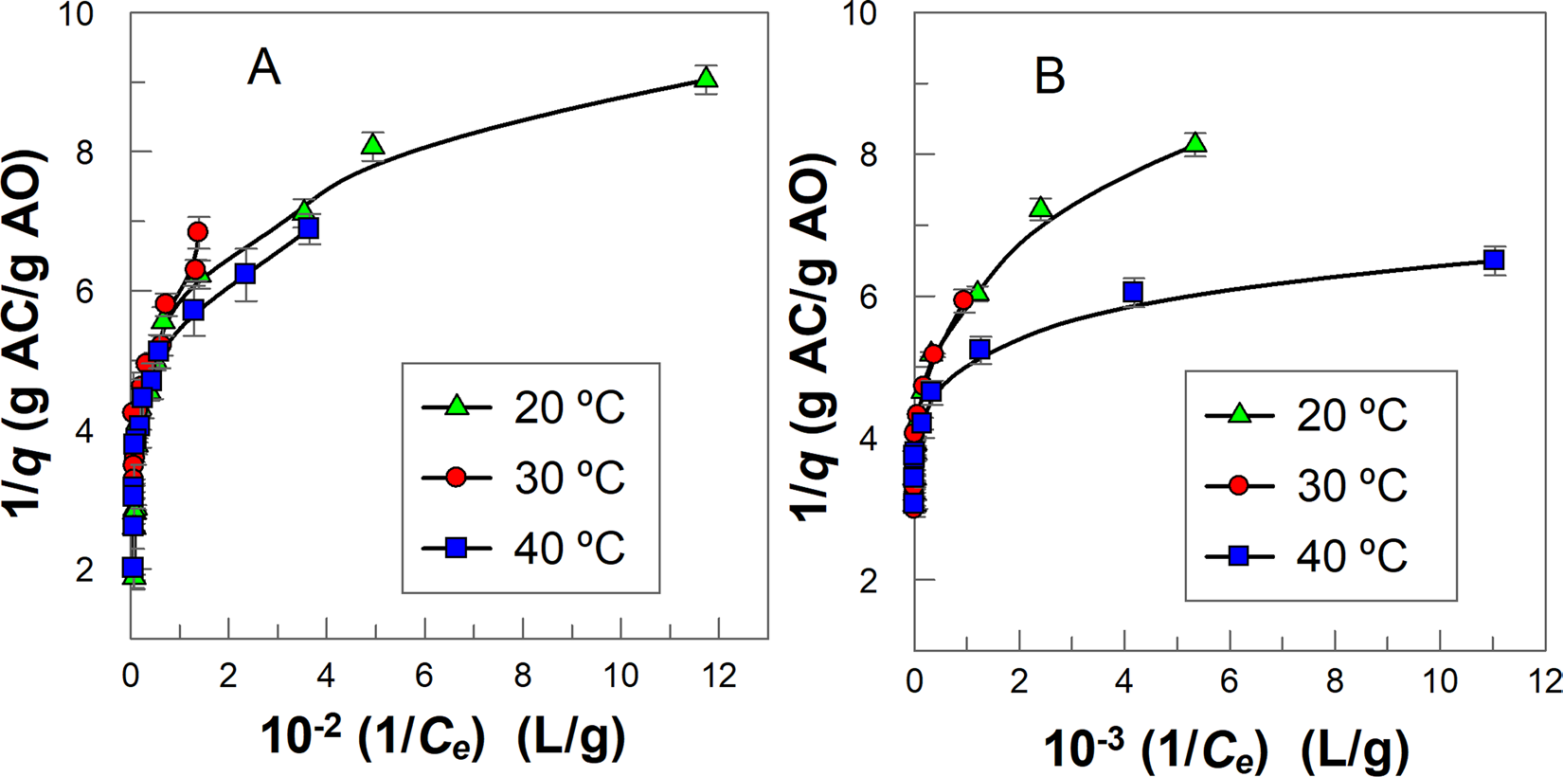
Langmuir adsorption isotherms for **(A)** GA and **(B)** PG at different temperatures. (*q* = adsorption capacity, *C*_*e*_ = equilibrium polyphenols concentration at the aqueous phase, g/L). The solid lines are drawn to aid the eye.

The obtained adsorption results were fitted to the linear form of the Langmuir isotherm (Eq. 2) at the investigated temperatures, Fig. 3. A non-linear plot was obtained over the studied amount of AC range. Therefore, results show that the model was not suitable for describing the adsorption of GA and PG on AC. Note, at any AC mass given, changes in the temperature lead to very small changes in the adsorptive capacity of GA and PG, differences less than 2% (e.g. in presence of 0.04 g AC, *q* = (0.219 ± 0.003) g GA/g AC at T = (20 ± 1) °C and *q* = (0.225 ± 0.002) g GA/g AC at T = (40 ± 1) °C).

### Freundlich isotherm

**Figure 4:**
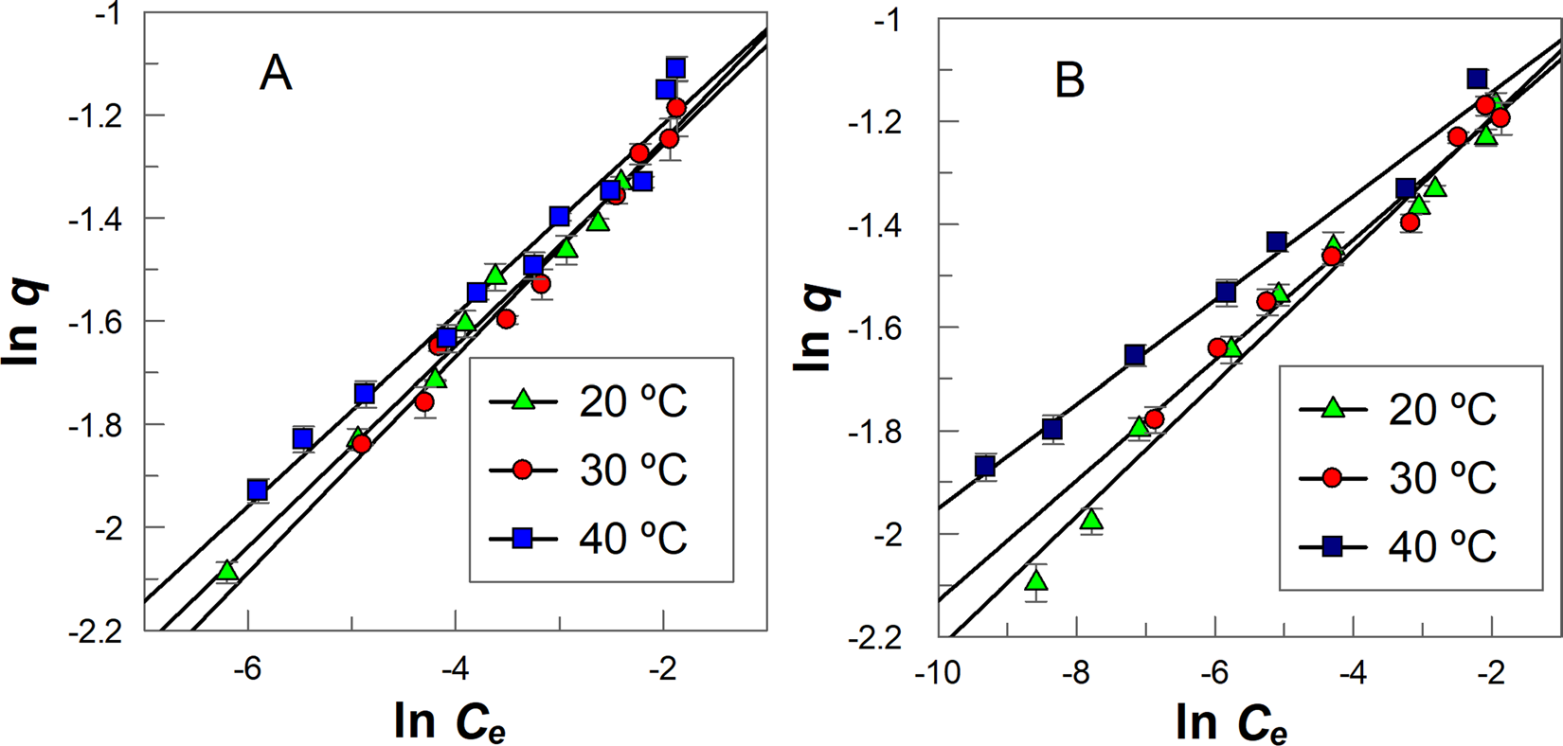
Freundlich adsorption isotherms for **(A)** GA and **(B)** PG at different temperatures, (T ± 1) °C. The solid lines are the theoretical lines obtained by fitting the experimental (ln *q*, ln *C*_*e*_) pair of data to Eq. 4.

The Freundlich isotherm considers adsorption on heterogeneous surfaces and gives a good interpretation of data in a narrow range of compound concentration. The Freundlich isotherm is described by Eq. (3), where *K*_*F*_ is a constant indicative of the relative adsorption capacity of the adsorbent and *n* is a constant indicative of the intensity of the adsorption (0 < *n* < 1, the adsorption process is favorable). Equation (3) can be rearranged to Eq. (4), which predicts that a plot of *ln q vs ln C*_*e*_ should be a straight line with an intercept value equal *n*. Figure 4 shows that this prediction is fulfilled and from slope and intercept values the Freundlich parameters (*K*_*F*_ and *n*) are shown in Table 2.

3 $$q={K}_{F}{C}_{e}^{n}$$

4 $$\mathrm{ln}\,q=\,\mathrm{ln}\,{K}_{F}+n\,\mathrm{ln}\,{C}_{e}$$

**Table 2:** Freundlich isotherm parameters for GA, PG and *Bryophyllum* aqueous extract. *K*_*F*_ is a measure of adsorption capacity, while *n* stands for the intensity of the adsorption. Results are expressed as value ± standard deviation. *R*^2^ is the correlation coefficient.

Antioxidant	(T ± 1) °C	10^2^ *K*_*F*_ (L/g)	10^2^ *n*	*R* ^2^
GA	20	41.91 ± 4.52	19.47 ± 0.97	0.995
	30	43.57 ± 4.29	20.96 ± 1.07	0.991
	40	42.86 ± 4.91	18.53 ± 1.10	0.984
PG	20	39.32 ± 3.61	12.92 ± 0.63	0.991
	30	38.16 ± 3.16	11,67 ± 0.70	0.990
	40	39.01 ± 3.99	10.10 ± 0.59	0.991
*Bryophyllum* extract	20	45.19 ± 3.61	39.62 ± 7.80	0.997

Results suggest that the adsorption process of GA and PG on AC exhibits a Freundlich behavior (correlation coefficients R^2^ > 0.99, Table 2), which is in line with literature reports^20–22^. The low *n* < 1 values indicate that GA and PG are adsorbed favorably. *n* and *K*_*F*_ values are independent of the temperature for both GA and PG, suggesting that the diffusion of GA and PG in the microporous structure of AC is temperature independent.

### Polyphenolic adsorption from *Bryophyllum* aqueous extract

Because we have shown that changes in temperature have only a minor effect on the adsorption processes of GA and PG, we only investigated the adsorption of *Bryophyllum* aqueous extract at a single T (T = 20 ± 1 °C) in order to minimize oxidation of the antioxidants, which may be appreciable at high temperatures^18,23,24^. The identification and quantification of the individual compounds of *Bryophyllum* aqueous extract was not done because this study is focused on adsorption of phenolic compounds from aqueous plant extracts, and we plan to use the mixture of antioxidants rather than the pure compounds for some future applications, for example, in studies aimed to analyze their efficiency in minimizing the oxidation of lipid-based functionalized foods (which will be part of future reports).

**Figure 5:**
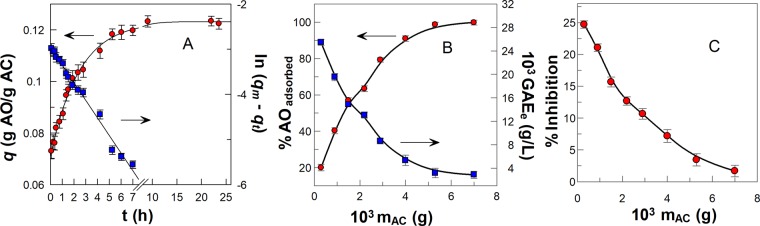
(**A**) Adsorption kinetic plot for polyphenols from *Bryophyllum* extract on AC at T = (20 ± 1) °C. The solid lines are the theoretical curves obtained by fitting the (*q*, time) or (ln (*q*_*m*_ − *q*_*t*_), time) pairs of data to the integrated first order equation. **(B)** Variation of the percentage of polyphenols from *Bryophyllum* extract adsorbed on AC (●) and polyphenol concentration, expressed as GAE_e_ (■), at the aqueous phase with increasing AC mass. **(C)** Changes in radical scavenging activity, expressed as the percentage of the inhibition of aqueous phase containing *Bryophyllum* extract, in presence of different AC amounts at T = (20 ± 1) °C. The solid lines in Figures (**B**,**C**) were drawn to aid the eye.

Figure 5A shows a typical kinetic plot illustrating the changes in the *q* values with time for *Bryophyllum* aqueous extract and the corresponding first-order plot. The obtained *k*_1_ value for adsorption rate constant of polyphenols from *Bryophyllum* aqueous extract is lower than that for GA and PG (Table 1), suggesting that their rate of diffusion across the external boundary layer and the internal pores of the AC is slower than that for GA and PG. The adsorption equilibrium is reached after 7 hours and it is maintained for, at least, 24 h.

The percentage of polyphenols from *Bryophyllum aqueous* extract adsorbed increased from ∼20% to >99% going from 0.3 mg to 7 mg of AC and, as a consequence, the polyphenols concentration in the aqueous phase, expressed as total gallic acid equivalents at the equilibrium GAE_e_ (g/L), decreases from 0.02 g/L to 0.003 g/L (i.e. ∼7 fold), Fig. 5B. Thus, >99% of polyphenols in the plant extracts were successfully adsorbed on the adsorbent surface by employing a relatively low AC mass.

Polyphenols from plant extract exhibited antioxidant activity toward the radical 2,2-diphenyl-1-pycrilhydrazyl (DPPH^•^), and the antioxidant efficiency of the polyphenols (expressed as %*Inhibition*) decreased from ∼25% to ∼2% in the aqueous phase with increasing AC mass, Fig. 5C, because most of the AOs were adsorbed in the AC surface.

**Figure 6:**
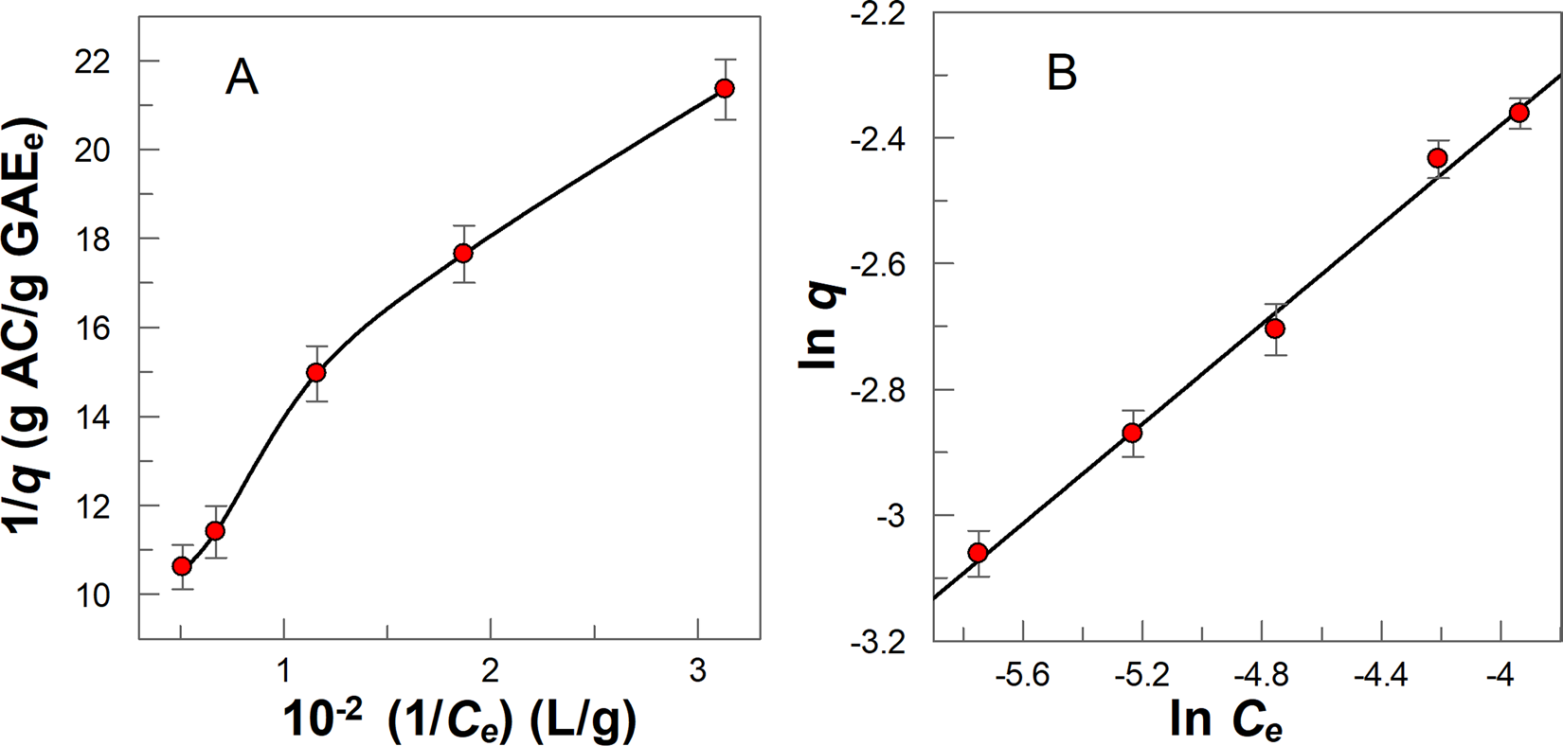
Langmuir **(A)** and Freundlich **(B)** adsorption isotherms for *Bryophyllum* aqueous extract at T = (20 ± 1) °C. *C*_*e*_ = equilibrium polyphenols concentration expressed as total gallic acid equivalents at the equilibrium, GAE_e_ (g/L). The solid line in Figure A was drawn to aid the eye, but that in Figure B is the theoretical curve line obtained by fitting the (ln *q*, ln *C*_*e*_) pair of data to the Eq. 4.

The experimental isotherms obtained for *Bryophyllum* aqueous extracts are displayed in Fig. 6. The equilibrium parameters were obtained using the linearized forms of Langmuir and Freundlich isotherms, Eqs (2) and (4), respectively, using the same set of experimental data. For instance, the straight line in Fig. 6B shows that the experimental data fits better the Freundlich isotherm than the Langmuir isotherm (Fig. 6A), suggesting that several layers of polyphenols may be adsorbed in the AC surface by physical forces. Values for the calculated parameters for the Freundlich isotherm and the corresponding correlation coefficients, *R*^2^, are indicated in Table 2.

According to our experimental results, the adsorption behavior for *Bryophyllum* aqueous extract is similar to pure compounds GA and PG (Figs 3, 4 and 6) since all of them fit the Freundlich adsorption isotherm. While *K*_*F*_ values, which represents an index of the adsorption capacity of AC, for GA, PG and the polyphenols from plant extracts are very similar (differences < 15%), the *n* values for the adsorption of polyphenols from plant extracts are significantly higher than those obtained for GA and PG (Table 2), following the order: *n*_PG_ < *n*_GA_ < *n*_plant extract_, with *n* < 1 in all cases (favorable adsorption process). Such an observation suggests that the solubility of antioxidants in aqueous solution seems to play a significant role on their adsorption process on AC.

## Conclusions

In summary, here we proposed a novel approach that combines the use of *in vitro* plant tissue culture and an effective and sustainable adsorption methodology for extraction of polyphenolic compounds from plant extracts. The *in vitro* culture of *Bryophyllum* individuals under aseptic conditions provides a controlled, pathogen-free biological system for the valuable production of bioactive compounds from plant sources. This *in vitro* methodology is a safe, simple and reproducible strategy with potential industrial and economical targets. The use of activated carbon provides an environmental friendly way of removing the polyphenols from the plant extracts.

The characterization adsorption of pure polyphenols on AC, such as GA and PG, confers insight about the adsorption behavior of the complex plant extracts. In this sense, the adsorption kinetics for GA and PG were fitted to the pseudo first order equation at the different temperatures with a good correlation coefficient and equilibrium was reached after 4 h of the contact time.

From equilibrium experiments, the Freundlich isotherm models were found to successfully predict the experimental data for both pure compounds and *Bryophyllum* aqueous extract. Since temperature did not affect the adsorption capacity of AC for studied antioxidants, adsorption of aqueous *Bryophyllum* extract was analyzed under reliable conditions, T = (20 ± 1) °C, which ensured a presumable protection against chemical oxidation of their polyphenolic constituents.

Results demonstrate that polyphenolic compounds from *Bryophyllum* aqueous extract can be successfully adsorbed on AC (>99% at 7 mg AC). Results may serve as starting point for the future application of AC on the recovery of antioxidants from plant extracts, by avoiding the typical disadvantages of traditional extraction and purification procedures. Some activated carbons are used in the food industry^25–28^, and so they may be used as food additives loaded with natural antioxidants from plant extracts. However, the desorption process may not be a simple task, and investigations on potential desorption processes, and on the use of AC loaded with antioxidants from plant extracts as additives in lipid-based products to, for instance, minimize the oxidation of lipids, are in progress and will be part of future reports.

## Experimental

### Materials

All chemicals were of the highest purity available and used as received. The AC was extra pure charcoal activated powder from Merck, Catalog No K49794484816 (BET specific Surface area (m^2^/g) 800; particle size (nm) 50–150, >80%; apparent density 150–440 kg/m^3^). Gallic acid monohydrate and propyl gallate were both 98.0% pure, purchased from Sigma-Aldrich and Fluka, respectively. Folin-Ciocalteu’s reagent was obtained by VWR Chemical. Sodium carbonate and 2,2-diphenyl-1-pycrilhydrazyl (DPPH^•^) were purchased from Sigma-Aldrich. Milli-Q grade water was employed in the preparation of aqueous solutions. All materials and culture media used for the establishment of *in vitro* culture were previously autoclaved at 98 kPa and T = (121 ± 1) °C for 20 minutes.

### Instrumentation

UV-VIS spectra and absorbance measurements were obtained on an Agilent 8453 spectrophotometer equipped with a cell carrier thermostated with water from a Julabo F12-ED bath and interfaced with a computer for data analyses and storage. An AND analytical balance with a precision of ± 0.00001 g was employed to weigh the required amounts of reactants. Aliquots of solutions were removed from the corresponding stock solutions with the aid of Socorex Acura micropipettes of 100 ± 1 μL and 1000 ± 5 μL.

### Plant material and *in vitro* culture establishment

Fully-developed epiphyllous buds from greenhouse-grown adult plants of *Bryophyllum* × *houghtonii* were excised from their mother plant and subjected to surface sterilization. Buds were maintained in non-sterile tap water over-night. Once washed, buds were gently dried with paper filter at room temperature prior to their surface disinfection, which was developed inside a laminar flow cabinet, under aseptic conditions. Explants were firstly treated with 70% (v/v) ethanol for one minute, followed by a wash with sterile distilled water and a subsequent disinfection step, using 0.4% (v/v) sodium hypochlorite with a few drops of Tween-20 for 10 minutes. Finally, explants were thoroughly washed with sterile distilled water and dried with paper filter to achieve the total removal of surface disinfection agents.

Once disinfected, buds were transferred to culture vessels containing 25 mL of MS medium (Murashige and Skoog basal culture media specifically designed for plant tissue culture)^29^ with half macronutrient concentration, supplemented with 3% (w/v) sucrose at pH = 5.8 and solidified with 0.8% (w/v) agar. The cultures were kept for 12 weeks in a growth chamber under a photoperiod of 16 h light and 8 h dark, at T = (24 ± 1) °C. Epiphyllous buds from grown plants were subcultured every 12 weeks into fresh medium. Aerial tissue collected from the third subculture was used for the subsequent plant extraction. No tissue injury nor necrosis were observed in cultured explants as a consequence of disinfection procedure, since plants grew healthy.

### Plant extraction

Aerial parts from adult *B*. × *houghtonii in vitro*-grown plants were collected and frozen at T = (− 20 ± 1) °C, prior to their lyophilization. Dried material was homogenized and 100 mg of obtained fine powder was extracted with 10 mL of water, vortexed and heated in a water bath at T = (60 ± 1) °C for 10 minutes, followed by a sonication period of 30 minutes. Samples were allowed to reach room temperature prior to sonication step. The supernatant was separated and the remaining plant material was re-extracted under the same conditions. Finally, the supernatants were combined and the extract was diluted at a final concentration of 2 g/L expressed in terms of the total gallic acid equivalents (GAE) and filtered through 0.45 µm PTFE membrane filters.

### Adsorption experiments

#### Adsorption of GA and PG

50 mL of antioxidant stock solution (0.2 g/L, pH = 2.0 adjusted with HCl) were incubated at different temperatures (T = 20–40 °C) with specific amounts (0.004–0.09 g) of AC and shaken in a thermostated orbital shaker -Incubator Heidolph 1000 orbital stirrer equipped with a Heidolph thermostat 1010 temperature control (which ensures a precision of  ± 1 °C), in the dark (195 rpm)-. In sets of experiment (different T), samples with no added AC were used as control to check the antioxidant stability. Bulk solutions were subsequently filtered through Whatman paper (73 g/m^2^). After completion of adsorption, a volume of ca 10 mL of supernatant was used to saturate the paper filter with polyphenols. This volume of filtrate was discarded. After that, another aliquot of the supernatant was filtered through the – previously saturated - filter paper and the filtered aqueous solution was used to make the corresponding measurements by UV spectrometry (λ = 271 nm). Results were expressed as the ratio between the amount of antioxidant (AO) adsorbed (in grams) and the mass (grams) of AC at equilibrium, *q* (g AO/g AC), given by Eq. (5), where *m*_*AO adsorbed*_ is the mass of AO adsorbed (g), *m*_*AC*_ is the mass of AC (g), *C*_*o*_ and *C*_*e*_ are the initial and equilibrium antioxidant concentrations (g/L), respectively, and *V* is the volume of bulk solution (L).

5 $$q=\frac{{m}_{AOadsorbed}}{{m}_{AC}}=\frac{({C}_{0}-{C}_{e})V}{{m}_{AC}}$$

The adsorption kinetics of GA and PG were monitored by measuring the amount of AOs adsorbed onto the AC (*q* value) at specific time intervals for 24 h, which was more than sufficient time to reach equilibrium. Values of observed rate constant, *k*_1_, were determined from the variation of *q* with time by fitting the (*q*, time) data points to the integrated first-order equation, Eq. (6), using a nonlinear least-squares method provided by a commercial computer program (GraFit 5.0.5). In Eq. (6), *q*_*m*_ is the maximum antioxidant adsorption (reached at t = ∞, g AO/g AC), *q*_*t*_ is the amount of adsorbed AO at time *t* (g AO/g AC), *k*_*1*_ is the pseudo-first order adsorption constant (h^-1^) and *t* is the adsorption time (h).

6 $${q}_{t}={q}_{m}\cdot (1-{e}^{-{k}_{1}t})$$

#### *Bryophyllum* extract adsorption

25 mL Erlenmeyer flasks containing 10 mL of an aqueous solution of plant extract (∼2 g/L of GAE) and specific amounts (0.003–0.015 g) of AC were placed in an orbital shaker (195 rpm) in the dark and allowed to reach thermal equilibrium for at least 24 h. The AC was separated as described above. Polyphenolic concentration of the aqueous plant extract, expressed as total gallic acid equivalents at the equilibrium, GAE_*e*_ (g/L) and antioxidant activity were determined by Folin-Ciocalteu’s and DPPH^•^ methods, respectively. The initial total phenolic content of *B*. × *houghtonii* aqueous extract was 0.028 g/L GAE.

### Antioxidant assays

#### Total phenolic content determination

Total phenolic content (TPC) was determined by the application of Folin-Ciocalteu´s reagent^30^. Briefly, 100 µL of sample were mixed with 200 µL of 10% (v/v) Folin-Ciocalteu’s reagent for 2 minutes at room temperature. Then, 800 µL of 0.7 M sodium carbonate were added and the reaction mixture was vortexed and incubated at T = (25 ± 1) °C in the dark for 2 h. The absorbance was measured at λ = 765 nm. A calibration curve was performed using GA as standard and the results were expressed as total gallic acid equivalents at the equilibrium, GAE_e_ (g/L). All determinations were carried out in triplicate.

#### Radical scavenging activity determination

Antioxidant activity was analyzed through the determination of radical scavenging activity (RSA), using the stable free-radical 2,2-diphenyl-1-pycrilhydrazyl (DPPH^•^). DPPH^•^ is a colored free-radical which is neutralized under the presence of antioxidants. Briefly, 2850 µL of the DPPH^•^ methanolic solution 110 µM were mixed with 150 µL of filtrates, vortexed and incubated at T = (25 ± 1) °C for 24 h. The diminution on DPPH^•^ signal was followed by UV spectrometry (λ = 517 nm) with the aid of a previously prepared calibration curve. RSA results were expressed as *%Inhibition* calculated as Eq. (7) where *A*_0_ and *A*_*e*_ are the absorbance values of DPPH^•^ in the absence and in the presence of polyphenols, respectively.

7 $$ \% Inhibition=\frac{({A}_{0}-{A}_{e})}{{A}_{0}}\times 100$$

## Data Availability

All data generated or analyzed during this study are included in this manuscript.
